# Coronary vasospasm during pulsed field focal ventricular ablation with solid tip catheter

**DOI:** 10.1093/europace/euaf226

**Published:** 2025-09-19

**Authors:** Kazuto Hayasaka, Petr Peichl, Nicoletta Ventrella, Josef Marek, Peter Štiavnický, Eva Borišicová, Jana Hašková, Predrag Stojadinovič, Robert Čihák, Dan Wichterle, Josef Kautzner

**Affiliations:** Department of Cardiology, Institute for Clinical and Experimental Medicine, Vídeňská 1958/9, Prague 140 00, Czechia; Department of Cardiology, Institute for Clinical and Experimental Medicine, Vídeňská 1958/9, Prague 140 00, Czechia; Department of Cardiology, Institute for Clinical and Experimental Medicine, Vídeňská 1958/9, Prague 140 00, Czechia; 2nd Department of Medicine—Department of Cardiovascular Medicine, Charles University Medical School I, Prague, Czechia; Department of Cardiology, Institute for Clinical and Experimental Medicine, Vídeňská 1958/9, Prague 140 00, Czechia; 2nd Department of Medicine—Department of Cardiovascular Medicine, Charles University Medical School I, Prague, Czechia; Department of Cardiology, Institute for Clinical and Experimental Medicine, Vídeňská 1958/9, Prague 140 00, Czechia; Department of Cardiology, Institute for Clinical and Experimental Medicine, Vídeňská 1958/9, Prague 140 00, Czechia; Department of Cardiology, Institute for Clinical and Experimental Medicine, Vídeňská 1958/9, Prague 140 00, Czechia; Department of Cardiology, Institute for Clinical and Experimental Medicine, Vídeňská 1958/9, Prague 140 00, Czechia; Department of Cardiology, Institute for Clinical and Experimental Medicine, Vídeňská 1958/9, Prague 140 00, Czechia; Department of Cardiology, Institute for Clinical and Experimental Medicine, Vídeňská 1958/9, Prague 140 00, Czechia; 2nd Department of Medicine—Department of Cardiovascular Medicine, Charles University Medical School I, Prague, Czechia; Department of Cardiology, Institute for Clinical and Experimental Medicine, Vídeňská 1958/9, Prague 140 00, Czechia

**Keywords:** Pulsed-field ablation, Ventricular arrhythmia, Coronary spasm, Irrigated-tip catheter, Coronary venous system

## Introduction

Coronary spasm is a recognized complication of pulsed-field ablation (PFA).^1^ Although most reports have focused on large-footprint systems, risks of PFA delivered via focal catheter are less known. The newly available CENTAURI generator (Cardiofocus) enables delivery of monopolar, biphasic PFA using compatible conventional ablation catheters and it has been successfully used for treatment of ventricular arrhythmias (VA).^2^ Recent reports indicate that focal PFA can be performed even from within the coronary venous system (CVS) without inducing spasm.^3^ However, we have encountered several cases of spasm following focal PFA. Our aim is to share insights from these experiences.

## Methods

The study included patients who underwent treatment for VA using CENTAURI system from May 2023 to June 2025. For navigation, the CARTO 3 mapping system (Biosense Webster) and intracardiac echocardiography (ICE) were utilized. A 3.5-mm irrigated-tip catheter (ThermoCool SmartTouch™, Biosense Webster) was used for both mapping and ablation. For PFA, 25A applications with a pulse width of 3.4 ms were delivered up to three times at each target site. Coronary angiography was conducted before and after each application to check for the presence of spasm. The distance between the catheter tip and coronary arteries were evaluated using biplane fluoroscopy or ICE.

## Results

Of 13 patients treated for VA using PFA near coronary arteries, vasospasm occurred in four patients (31%). The ablation sites were located in the left ventricular outflow tract involving the great cardiac vein (GCV) in 11 patients, the LV posterior wall in one patient, and the right ventricular outflow tract (RVOT) in one patient. Regarding the distance between the catheter and the coronary artery, in the four cases with spasm the mean distance was 0.75 ± 0.65 mm, whereas in the nine cases without spasm it was 3.8 ± 2.5 mm.

### Case 1

**Figure 1:**
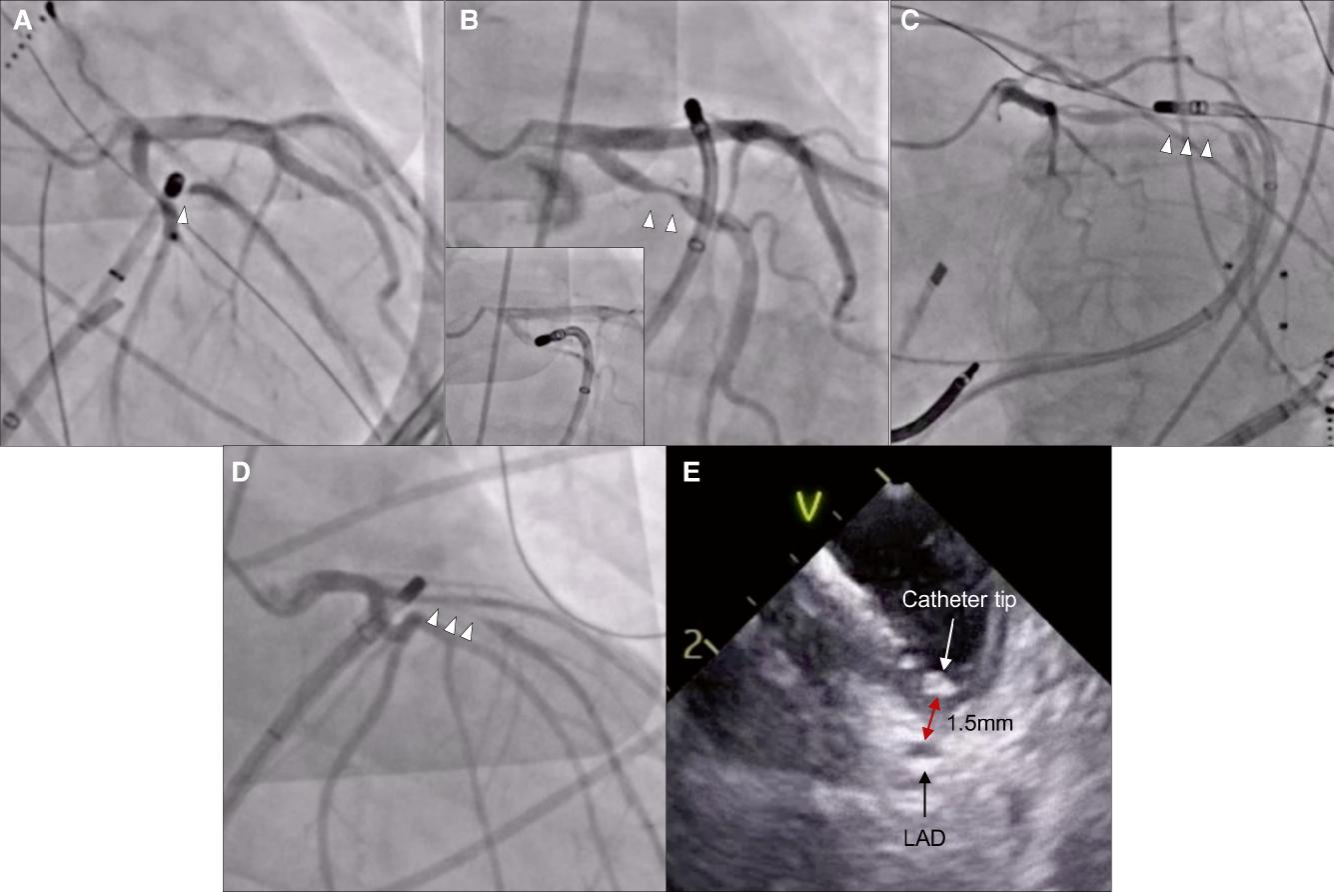
Spasm cases and the spatial relationship and distance between the catheter tip and coronary arteries. (*A*) The angiography showed 90% spasm in left circumflex artery (LCX) #12. The catheter was positioned directly over the coronary artery. (*B*) Pulsed-field ablation (PFA) resulted in 90% LCX #11 spasm. The small window showed that the catheter crossed the artery at a distance of approximately 1 mm. (*C*) The catheter was parallel to the artery over a relatively long distance. (*D*) Left descending artery #6 developed 99% spasm by PFA from right ventricular outflow tract. (*E*) Intracardiac echography showed that the catheter was close to the artery during PFA. LAD, left anterior descending artery.

A 51-year-old woman with prior failed radiofrequency ablation (RFA) for summit-origin premature ventricular contractions (PVC) underwent a redo procedure. Pulsed-field ablation was performed in the distal GCV, where the earliest activation was recorded 32 ms pre-QRS. Post-ablation angiography showed 90% spasm of the left circumflex artery (LCX) without ST-segment changes (*Figure 1A*). During PFA, the catheter was positioned directly over the coronary artery. The vessel diameter returned to baseline one hour after intracoronary nitrate (1 mg) administration. Additional RFA and PFA from the left coronary cusp (LCC) successfully suppressed the PVC without further spasm, and no recurrence has been observed during the 3-month follow-up.

### Case 2

A 57-year-old woman with a 21% PVC burden from the left ventricular summit underwent ablation. Early activation was detected at the LCC, but RFA at this site was ineffective. At the GCV, 15 ms pre-QRS prompted the delivery of PFA. Despite intranasal nitrate administration (0.8 mg), the patient developed 90% spasm of LCX after PFA (*Figure 1B*) without ST-segment changes. The coronary spasm resolved within 10 min following intracoronary nitrate (0.5 mg) administration. The distance between the catheter and the artery was 1 mm. Subsequent RF and PF above the pulmonary valve eliminated the PVC, no recurrence was observed during the 3-month follow-up.

### Case 3

An 80-year-old woman with dilated cardiomyopathy experienced recurrent ventricular tachycardia (VT) storm despite seven prior ablation procedures. During substrate mapping, low-voltage areas were identified in the lateral wall and summit. Coronary angiography demonstrated 25–50% pre-existing stenosis on LCX. Pulsed-field and RF were applied endocardially and epicardially to the basal superior left ventricle via the GCV, rendering VT non-inducible. However, a 75% spasm was observed in LCX (*Figure 1C*) with pre-existing stenosis. The distance between the catheter and the artery was 0.5 mm. Intracoronary administration of a total of 5 mg nitrate resolved the spasm. Despite acute VT non-inducibility, VT has recurred, and adjunctive pharmacological therapy was administered.

### Case 4

A 15-year-old boy who had previously undergone outflow tract PVC ablations experienced recurrence with a burden of 46%, prompting a redo procedure. The earliest activation was observed in the anteroseptal region of the RVOT. Initial angiography suggested a safe distance from the coronary arteries; however, additional PFA at a more inferior position induced 99% spasm in the left anterior descending artery (LAD) without ST-segment changes (*Figure 1D*). Intracardiac echocardiography revealed the catheter was 1.5 mm from the artery (*Figure 1E*). The spasm resolved 30 min after intracoronary nitrate administration with a total dose of 12.5 mg. Premature ventricular contractions were successfully eliminated, with no further recurrence at the 1-year follow-up.

## Discussion

In PFA, lesion formation is determined by catheter electrode configuration. With a bipolar electrode configuration, current loss occurs into the blood pool as the energy is transmitted between the two electrodes, resulting in relatively shallow lesions.^4^ In contrast, the CENTAURI system employs a unipolar PF system, in which one electrode is positioned at a site distant from the other, allowing the energy to traverse the myocardium towards the return electrode and thereby creating deeper lesions. Animal studies reported depths of 8.2 ± 1.6 mm^5^ after unipolar PFA, indicating potential benefit in cases resistant to bipolar PF or RF ablation. Its ability to deliver energy in high-impedance regions, such as a distal GCV, also broadens applicability. Given these features, wider use may be expected; however, coronary spasm remains a concern, and the risk may be greater than with bipolar PF due to deeper lesion formation. Reports of spasm during VA ablation using focal PFA have been limited and included a case of mild spasm on angiography.^6^ However, as demonstrated in the current series, clinically significant spasm may occur easily with focal PFA, especially when the catheter tip is positioned directly on the coronary artery, and such may not be accompanied by prominent ST-segment changes. This is noteworthy, as prior reports primarily focused on PFA for atrial arrhythmias.^7,8^ These findings underscore the importance of coronary angiography before and after ablation, even with focal PFA delivered by solid tip catheter.

While prophylactic nitrate administration is often considered, its effectiveness remains uncertain for a focal PF system. A previous study using high-dose intravenous nitroglycerin successfully prevented spasm during cavotricuspid isthmus ablation (CTI) with a multispline catheter.^9^ However, the catheter characteristics in that study differ from those used in a focal PF system, which may limit generalizability. In our series, nitrates were administered to only one patient, limiting our ability to conclude their preventive efficacy. Another study reported spasm despite 400 μg of intravenous nitroglycerin prior to focal PFA for CTI.^10^ Moreover, spasm in the proximal LAD or LCX during VA ablation could compromise a significantly larger portion of myocardium compared to spasm in the distal right coronary artery during flutter ablation, emphasizing the clinical relevance of this complication. Further research is warranted to determine the optimal nitrate strategy for use with focal PFA.

## Conclusion

In the present study, we observed coronary spasm during PFA from the CVS and RVOT. These findings indicate that such complications can occur with focal PFA, warranting further attention.

## Data Availability

The data underlying this article will be shared on reasonable request to the corresponding author.
